# Pharmacology of airways and vessels in lung slices *in situ*: role of endogenous dilator hormones

**DOI:** 10.1186/1465-9921-7-111

**Published:** 2006-08-21

**Authors:** L Moreno, F Perez-Vizcaino, L Harrington, R Faro, G Sturton, PJ Barnes, JA Mitchell

**Affiliations:** 1 Cardiothoracic Pharmacology, and Airway Disease Section, National Heart & Lung Institute, Imperial College London, Dovehouse Street SW3 6LY, UK

## Abstract

Small airway and vessels play a critical role in chronic airway and pulmonary vascular diseases, but their pharmacology has not been well characterised. We have studied airway and vascular responses in rat lung slices and separately *in vitro *using myography. In lung slices, under basal conditions, acetylcholine contracted airways, but had no vascular effect. The thromboxane mimetic, U46619 contracted both vessels and airways. In the presence of U46619, acetylcholine dilated vessels, but further contracted airways, an effect that was blocked by the nitric oxide synthase inhibitor L-N^G^-nitro-L-arginine or apamin plus charybdotoxin, which inhibit endothelial-derived hyperpolarising factor. Airway responses in lung slices were unaffected by L-N^G^nitro-L-arginine methyl ester, indomethacin or apamin plus charybdotoxin. By contrast, apamin plus charybdotoxin contracted bronchi studied in isolation. Our observations are the first to identify mechanisms of endothelium dependent dilations in precision cut lung slices and the potential for transverse hormonal communication between airways and vessels.

## Background

In mammals the lung is made up of conducting airways that carry air to the alveoli, the gas-exchanging units of the lung. The airways branch from the hilum towards the periphery. From the trachea to the terminal airways the diameter decreases but there is a gradual increase in cross-sectional area, because of the increase in number of airways [1]. In the adult lung the pulmonary arteries run alongside the airways, branching with them and decreasing in diameter. They supply blood to the capillary area closely matching that of the alveolar surface area. The pulmonary veins drain the capillary bed and though they do not run alongside the airways they have an equivalent number of branches to the arteries. The close relationship of the blood vessels and the airways is found throughout the lung. However our understanding how they function in parallel *in situ *is incomplete.

Acetylcholine dilates blood vessels [2] via activation of the endothelium and the subsequent release of NO, prostacyclin and endothelial-derived hyperpolarizing factor (EDHF), [3]. Acetylcholine constricts airways through activation of muscarinic receptors on airway smooth muscle cells [4]. However, it has been suggested that a bronchodilator is released by the epithelium and that this 'factor' could be NO [5]. Tonic responses of airways or pulmonary vessels are general studied separately, in isolation using organ baths. In addition, some groups have investigated tonic responses in airways [6-9]) or pulmonary vessels [10,11]*in situ *in whole lung slices using agonists. Methods applied to study airway and vascular responses in the whole lung slices rely on the infusion of a scaffold material such as agarose to facilitate the efficient sectioning of the tissue. Functional endothelial-dependent responses have been demonstrated in arteries and veins of guinea-pig lung slices, although comparisons with airway responses have not been made [12]. There are no studies in which responses in airways and vessels have been monitored simultaneously and where the role of endogenous dilator hormones (e.g. NO) released by either vascular endothelium, or airway epithelium, in responses have been addressed. In the current study we have measured the contractile and relaxant responses of airways and vessels *in situ *in whole lung slices using video microscopy [13]. Acetylcholine was added to the tissue in the presence and absence of a constrictor agent. The respective roles of NO, prostacyclin or EDHF in airway or vascular responses were addressed by pharmacological inhibitors. Finally, in each case, we have compared responses of vessels and airways in whole lung slices with those obtained using isolated structures in *in vitro *using wire myographs.

## 2. Materials and methods

### 2.1. Preparation of lung slices

Lungs were taken from 6–8 week-old (230–270 g) female Wistar rats and lung slices prepared as previously described [13]. All the animals used in this project were maintained and killed in accordance with The European Community guidelines for the use of experimental animals.

The animals were killed by lethal exposure to CO_2_, trachea was cannulated and the animals were exsanguinated by cutting the vena cava inferior. A small vertical cut into the diaphragm was made to collapse the lungs, followed by immediate instillation of 15 ml of 2% agarose (low melting point agarose) solution into the airways. After the agarose had cooled to 4°C, tissue cores were prepared by advancing a rotating, sharpened metal tube (diameter 8 mm) longitudinally. From these cores, tissue slices (250 μm) were prepared using a Krumdieck tissue slicer (Alabama Research and Development, Munford, AL, USA).

These slices were examined with an inverted microscope and those that contained at least one cross section of a vessel or an airway were placed in a 12 wells plate containing 1 ml of Dulbecco's modified Eagle's Medium (DMEM) supplemented with 100 units.ml^-1 ^penicillin, 0.1 mg.ml^-1 ^streptomycin, 4 mM L-glutamine and 2.5 μg.ml^-1 ^amphotericin B and incubated overnight on a roller system housed in a humidified incubator (37°C, 5% CO_2_-95% air). Medium was changed every 45 minutes for the first 3 hours. Sections of lung containing 2/3^rd ^order airways and vessels were taken in order to parallel the structures studied in isolation using the myographs (see below).

### 2.2. Image acquisition

Incubation and observation of slices was carried on an incubator chamber (PCLS-Bath Type 847, Hugo Sachs elektronik, Harvard Apparatus GmgH) containing 0.4 ml complete DMEM placed on the stage of a microscope (Nikon SMZ-U) and warmed to 37°C.

Arteries and airways were identified and imaged with a video camera (Image Associates, UK). To distinguish arteries from veins, we used criteria similar to those described previously [11]: 1) The arteries usually accompanied airways, whereas veins where at a distance from them, and 2) arterial walls had a thick media and their inner lining was slightly wrinkled, whereas veins were thinner and wrinkles were inconspicuous.

### 2.3. Experimental design

After preincubation for 5 minutes with 0.5 ml of DMEM, the first image was acquired ("baseline image"). Then, the liquid was removed and fresh medium added containing acetylcholine (10^-5 ^M). The slice was imaged every 20 seconds for 6 minutes followed by a wash step which led to a return to baseline. Then airways and vessels were precontracted with the thromboxane analogue 9,11-Dideoxy-11α, 9α-epoxymethanoprostaglandin F_2α _(U46619, 10^-7 ^M) and again images recorded every 20 seconds for 6 minutes. Acetylcholine (10^-5 ^M) was then added for 5 min, with images recorded each 20 sec. In some experiments acetylcholine was added to U46619 constricted airways and vessels in the presence of the nitric oxide synthase (NOS) inhibitor L-N^G^nitro-L-arginine methyl ester (L-NAME; 10^-3 ^M), the combined cyclo-oxygenase-1/cyclo-oxygenase-2 inhibitor indomethacin (10^-5 ^M) or the combination of apamin and charybdotoxin (5 × 10^-7 ^M and 10^-7 ^M) which together inhibit EDHF release (18). For these experiments incubations were continued for 10 minutes with images recorded each 20 seconds as above.

### 2.4. Image analysis

The images were analysed using an image analysis program (ZEISS KS 300 3.0). The luminal area was taken as the area enclosed by the epithelial luminal border and was quantified after setting the appropriate threshold value.

Baseline area was defined as 100%. The responses of arteries and airways were calculated as a percentage of baseline area using the equation: Response = residual area after drug/baseline area X 100. Thus a 0% response indicated complete luminal closure and 100% indicated no effect.

### 2.5. Myography

In lungs from separate animals, second- to third-order branch pulmonary arteries or bronchi were isolated from the lungs of female Wistar rats and placed into modified Krebs buffer (composition in 10^-3 ^M): NaCl 119, KCl 4.7, CaCl_2_, 2.5, MgSO_4 _1.17, NaHCO_3 _25, KH_2_PO_4 _1.18, EDTA 0.027 and glucose 5.5.

Pulmonary artery and bronchi were dissected our of fresh lungs and cut into small segments and mounted in a four channel Mulvanny-Halpern myograph under normalised tension (7.5 kPa) [14]. The segments were first challenged with high potassium solution (composition in 10^-3 ^M: KCl 123.7, CaCl_2 _2.5, MgSO_4 _1.17, NaHCO_3 _25, KH_2_PO_4 _1.18, EDTA 0.027 and glucose 5.5). Tissues were then washed and incubated once again in Krebs buffer.

Concentration-response curves to either U46619 (10^-9 ^to 10^-6 ^M) or acetylcholine (10^-8 ^to 10^-5 ^M) were then carried out and contractile responses in both airways or vessels recorded and represented as active effective pressure (AEP; Kpa; mN/mm^2^), calculated by the following equations: ΔT = ΔF/2x segment length; AET = ΔT/vessel radius where ΔT represent active wall tension and ΔF represents active force response measured in mN. Airways or vessels were then pre-contracted with an EC_80 _concentration of U46619 and acetylcholine added (10^-5 ^M). Responses were allowed to plateau before individual inhibitors of the NO, prostacyclin or EDRF pathways add, these were L-NAME (10^-3 ^M), indomethacin (10^-5 ^M) or apamin (5 × 10^-7 ^M) plus charybdotoxin (10^-7 ^M), [18] respectively.

Relaxant responses were calculated as a percentage of U46619-induced tone. Data are given as the mean ± SEM.

### 2.7. Materials

All drugs were purchased from Sigma Gilligham, Dorset, UK. Acetylcholine and indomethacin were freshly prepared each day in aqueous and ethanol solutions, respectively. U46619 was prepared in high concentration "stock" solution dissolved in ethanol and was stored at -80°C until used.

### 2.8. Statistics

Data was analysed using the appropriate tests and GraphPad Software. T-tests, one-way analysis of variance or one-sample T-test for normalised data was used as described in the text or in the figure legends.

## 3. Results

### 3.1. Effects of acetylcholine and U46619 on airway and vascular responses in precision cut lung slices in situ

**Figure 1:**
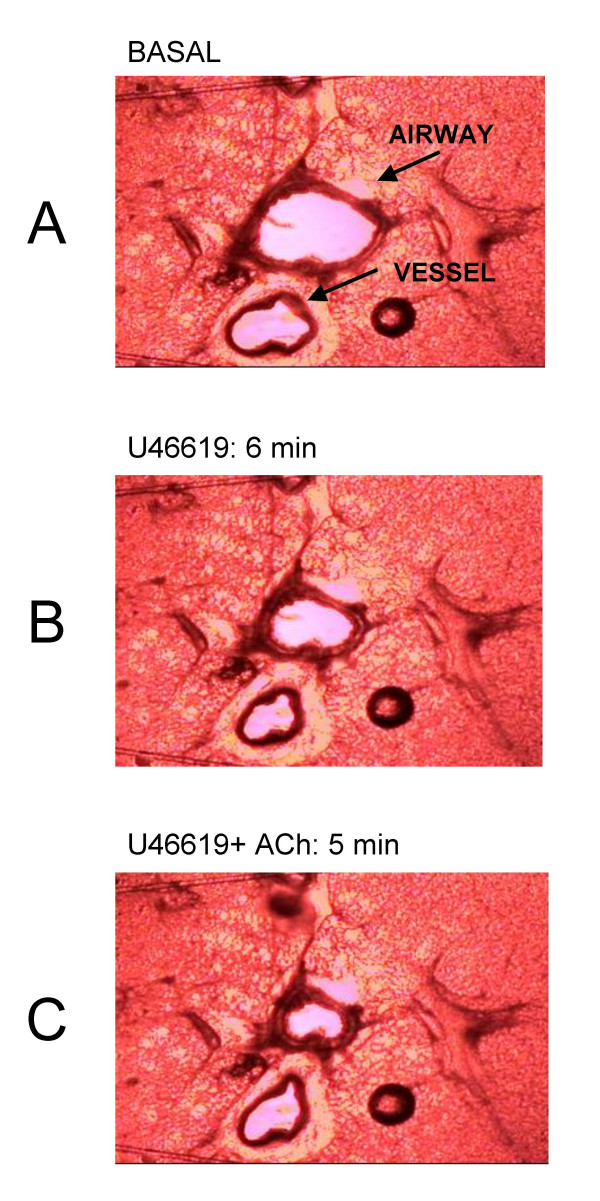
Effects of U46619 (10^-7 ^M) and acetylcholine (Ach; 10^-5 ^M) on internal luminal diameter of airway and vessels in whole precision cut lung slices. A; Tissue under control (basal conditions) bathed in medium alone. B; Tissue after 6 min stimulation with U46619. C; Tissue after 5 min stimulation with U46619 and acetylcholine. The images are representative of those used in the pooled data shown in Figure 2.

**Figure 2:**
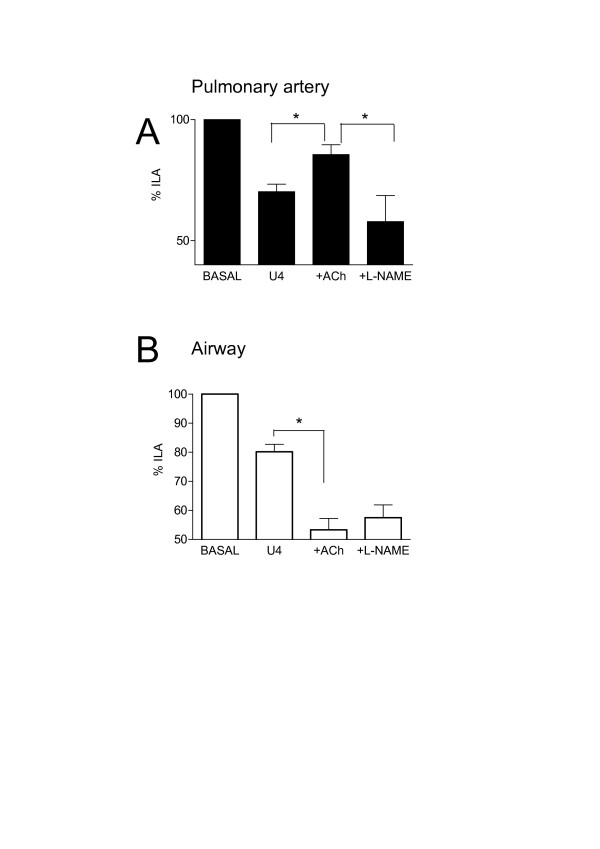
Effects of U46619 (U4; 10^-7 ^M) and acetylcholine (Ach; 10^-5 ^M) in the presence or absence of L-N^G^-nitro-L-arginine methyl ester (L-NAME) on internal luminal diameter of airway and vessels in whole precision cut lung slices. Panel A shows responses in pulmonary artery and panel B shows responses in bronchi. Measurements were made under basal conditions or after stimulation with U46619 (U4; 10^-7 ^M) or acetylcholine (Ach; 10^-5 ^M) or U4, plus Ach in the presence of L-NAME. The results are the mean +/- the S.E.M. for n = 4–6 experiments. A p-value of < 0.05 was taken as statistically significant, calculated using one sample t-test and denoted by *.

**Figure 3:**
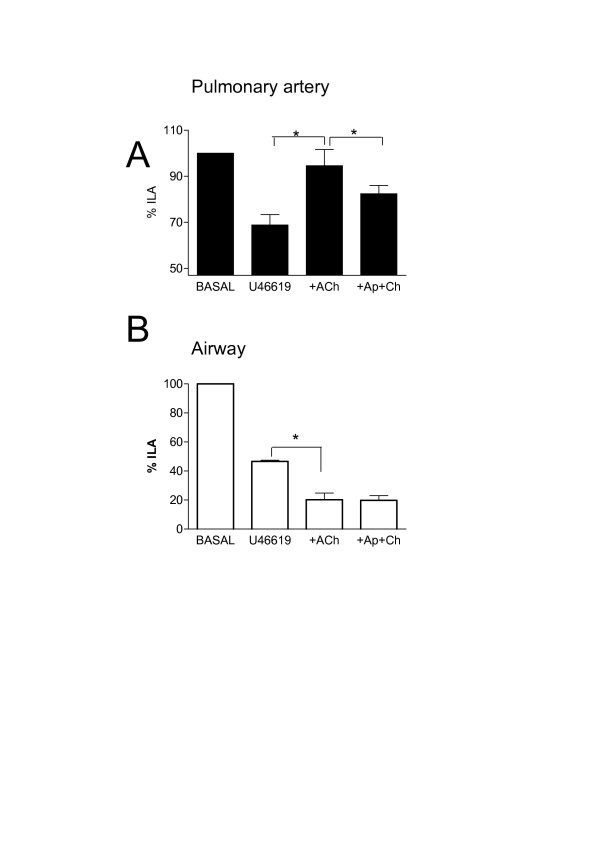
Effects of U46619 (10^-7 ^M) and acetylcholine (Ach; 10^-5 ^M), in the presence or absence of apamin (Ap, 5 × 10^-7^) plus charybdotoxin (Ch, 10^-7 ^M), (Ap+Ch), on internal luminal diameter of airway and vessels in whole precision cut lung slices. Panel A shows responses in pulmonary artery and panel B shows responses in bronchi. Measurements were made under basal conditions or after stimulation with U46619 (U4; 10^-7 ^M) or acetylcholine (Ach; 10^-5 ^M) or U4, plus Ach in the presence of Ap plus Ch. The results are the mean +/- the S.E.M for n = 3 experiments. A p-value of < 0.05 was taken as statistically significant, calculated using ANOVA and denoted by *.

Acetylcholine contracted airways (10^-5 ^M; -29.4 ± 7.3%) but had no significant effect (103.7 ± 1.7%) on 'basal' pulmonary vessel luminal area (n = 6). The thromboxane mimetic U46619 (10^-7 ^M) contracted both vessels (-37.8 ± 0.7%) and airways (-39.5 ± 1.9%) (Figure 1 and 2). Furthermore, in the presence of U46619, acetylcholine (10^-5 ^M) dilated the vessel but further contracted the airway (Figure 1 and 2). In separate experiments it was found that L-NAME (10^-3 ^M, % control; airway, 81.9 ± 7.4%: vessel 95.1 ± 6.6), indomethacin (10^-5 ^M; airway, 100.8 ± 7.63: vessel 92.4 ± 12.64) or apamin plus charybdotoxin (5 × 10^-7 ^M and 10^-7 ^M; airway, 96.3 ± 7.71; vessel, 89.1 ± 5.2) had no significant effect (using one-sample t-test; GraphPad) on basal airway or vascular tone (n = 4). However, L-NAME and the combination of apamin (5 × 10^-7 ^M) plus charybdotoxin (10^-7 ^M) blocked the vasodilator effects of acetylcholine in pre-constricted pulmonary vessels in lung slices (Figure 2 and Figure 3). Neither L-NAME nor apamin plus charybdotoxin affected contractile responses to U46619 or acetylcholine in airways in lung slices (Figure 2 and Figure 3). Indomethacin (10^-5 ^M) had no effect on any responses in either airway or vessel structures in the lung slice.

### 3.2. Effects of U46619 and ACh-induced responses in isolated airways and pulmonary arteries preparations in vitro

**Figure 4:**
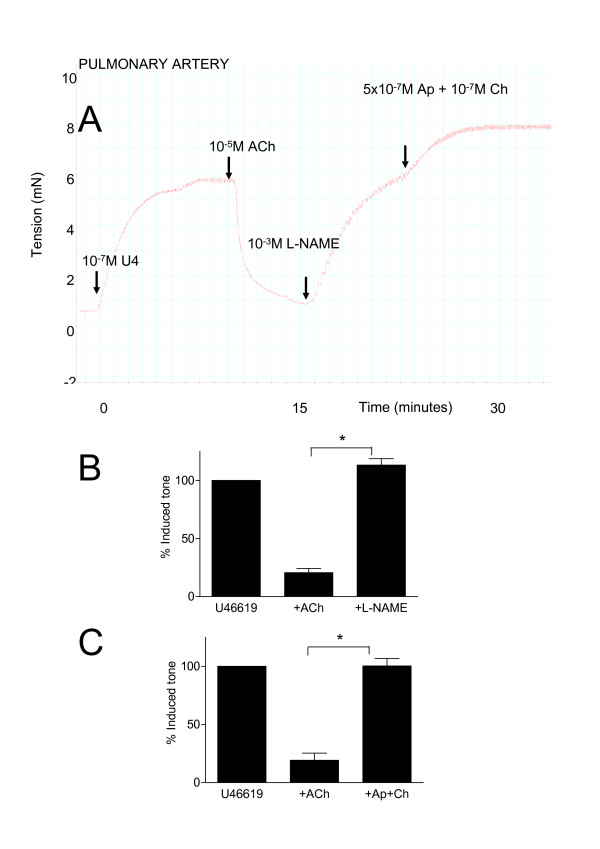
Characterisation of the dilator effects of acetylcholine (ACh; 10^-5 ^M) in isolated pulmonary artery. Panel A shows an original recording from a typical experiment. After equilibration, vessels were contracted with U46619 (10^-7 ^M), at plateau ACh was added and immediate dilation occurred. At plateau either L-N^G^-nitro-L-arginine methyl ester (L-NAME; 10^-3 ^M) or apamin (5 × 10^-7 ^M) plus charybdotoxin (10^-7 ^M) was added. Finally where L-NAME had been added, apamin plus charybdotoxin were added and visa versa. Panels B and C show pooled data from several experiments where L-NAME or apamin plus charybdotoxin were added respectively. The data is expressed as the mean percentage of induced tone +/- the S.E.M and comprised of n = 4–6 experiments. A p-value of < 0.05 was taken as statistically significant, calculated by t-test and denoted by *.

**Figure 5:**
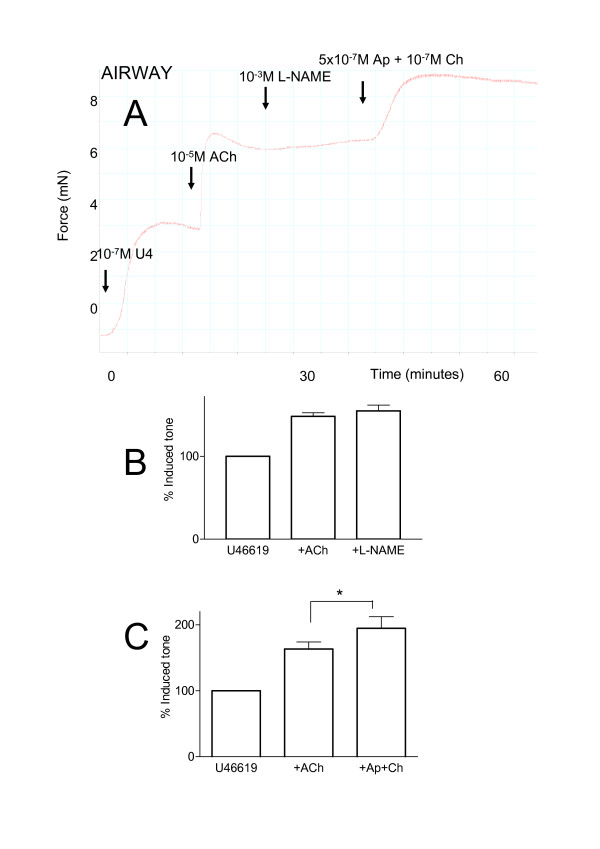
Characterisation of the effects of acetylcholine (ACh; 10^-5 ^M) in isolated pulmonary bronchi pre-constricted with U46619 (U4; 10^-7 ^M). Panel A shows an original recording from a typical experiment. After equilibration, bronchi were contracted with U46619 (10^-7 ^M), at plateau ACh was added and further contraction was observed. At plateau either L-N^G^-nitro-L-arginine methyl ester (L-NAME; 10^-3 ^M) or apamin (5 × 10^-7 ^M) plus charybdotoxin (10^-7 ^M) was added, once a further plateau was achieved apamin plus charybdotoxin or L-NAME were added respectively. Panels B and C show pooled data from several experiments where L-NAME or apamin plus charybdotoxin were added individually. The data is expresses as the mean percentage of U46619-induced tone +/- the S.E.M and comprised of n = 4–5 experiments. A p-value of < 0.05 was taken as statistically significant, calculated by t-test and denoted by *.

U46619 (10^-9 ^to 10^-6 ^M) induced concentration-dependent contractions in either bronchi or pulmonary arteries *in vitro *mounted in myographs (Emax 3.684 ± 0.877 mN and 5.306 ± 0.476 mN respectively). For both tissues 10^-7 ^M of U46619 represented an approximate EC_80 _concentration for contraction. Acetylcholine (10^-8 ^to 10^-6 ^M) induced concentration dependent contractions of bronchi, but had no effect of pulmonary artery preparations (0.355 ± 0.213 mN for 10^-6 ^M acetylcholine). When pulmonary vessels were pre-contracted with U46619 (10^-7 ^M) acetylcholine induced an immediate and profound and stable vasodilator response (Figure 4). When acetylcholine was added to airway tissue, pre-contracted with U46619, a further contraction was seen (Figure 5). When L-N^G^-nitro-L-arginine methyl ester (10^-3 ^M) was added to the pre-constricted vessels, stimulated with acetylcholine, it induced a rapid reversal of the dilator response (Figure 4). Similarly, when apamin (5 × 10^-7 ^M), plus charybdotoxin (10^-7 ^M), was added in the same way the combination of drugs completely reversed acetylcholine induced vasodilatation (Figure 4). Apamin plus charybdotoxin had no effect on basal tone in vessels (0% of basal tone). By contrast to results obtained in whole lung slices, the combination of apamin plus charybdotoxin further contracted airways stimulated with U46619 and acetylcholine (Figure 5).

## 4. Discussion

In the adult lung the pulmonary arteries run alongside the airways, branching with them and decreasing in diameter. Indeed, the airways and the vessels share an area of common interstitia which may allow transverse communication between the structures. It is therefore important to study vascular and airway responses in parallel and *in situ*. Previously this has been achieved using lung slices viewed using a microscope. However the relationship between airway and vascular responses to endogenous mediators released by the endothelium (or epithelium) has not previously been addressed.

Acetylcholine dilates blood vessels [2] via activation of the endothelium and the subsequent release of NO, prostacyclin and EDHF [3]. Similarly, whilst acetylcholine constricts airways via an action on the smooth muscle, [4] it can also, as in vessels, activate the lining cells -namely the epithelium, to release a bronchodilator substance [15,16]. The identity of epithelium-derived relaxing factor is unknown [17], although NO has been implicated [5]. In lung slices, we found that, under basal conditions, acetylcholine constricted airways, but had no effect on the adjacent pulmonary artery. For blood vessels to dilate, they first need to be constricted. Thus, these observations suggest that in lung slices either pulmonary vessels have no intrinsic tone or that the endothelium is not functional. When the thromboxane mimetic, U46619 was added to the lung slices both the airway and the pulmonary artery contracted. Under these conditions, the subsequent addition of acetylcholine produced significant dilator responses in the artery, but not in the airway of lung slices. These observations are consistent with what we found using isolated pulmonary arteries and airways *in vitro*. In lung slices, the dilator effects of acetylcholine on U46619 constriction tissue was blocked by L-NAME or by the combination of apamin plus charybdotoxin. L-NAME is a highly selective inhibitor of the NOS family. It inhibits all forms of NOS (NOSI, NOSII and NOSIII). In blood vessels, the release of NO induced by activation of the endothelium by agents such as acetylcholine (as used in this study) are always mediated by constitutive forms of NOS (principally NOSIII). NO release by NOSII is independent of calcium, and so would not by a stimulus such as acetylcholine. We can therefore, safely conclude that in lung slices the inhibitory effects we see with L-NAME are via the selective inhibition of NOSIII in the endothelium. Our results showing that the combination of apamin plus charybdotoxin (inhibitors of small and intermediate potassium dependent calcium channels respectively) also inhibited acetylcholine induced vasodilatation in vessels in the precision cut lung slice strongly suggest that EDHF is also release by these structures. Whilst others have suggested that pulmonary vessels *in vitro *release EDHF along with NO to mediate endothelium dependent dilator responses, we are the first to show this occurs *in situ*. This is an important point because the role of EDHF in vascular responses is contentious and not always demonstrable but instead is highly dependent upon the experimental conditions applied. These observations show that the mechanism of endothelium dependent dilation in lung vessels in precision cut lung sections is mediated by NO and EDHF. L-NAME had no effect on airway responses to acetylcholine in the absence or presence of U46619. Similarly apamin plus charybdotoxin did not influence airway responses in whole lung slices.

Results obtained in lung slices *in situ *were largely paralleled by responses of isolated pulmonary artery or bronchi *in vitro*. In isolated pulmonary artery preparations, the vasodilator effects of acetylcholine were unaffected by indomethacin, suggesting that prostacyclin release was not involved in vasodilatation of rat pulmonary artery. In contrast, in guinea-pig lung slices, indomethacin increased dilator responses, possibly via the inhibition of thromboxane release [11].

In isolated preparations acetylcholine had no relaxant effect on U46619-constricted airways. Indeed, as was seen in whole lung slices, acetylcholine further contracted U46619 constricted airways. Interestingly we found that by contrast to results in whole lung slices, the combination of apamin plus charybdotoxin further contracted isolated bronchi, pre-contracted with U46619 and acetylcholine. It is not clear why apamin plus charybdotoxin should have constrictor effects (albeit small and indirect) on isolated airway tissue, but not on airways *in situ*, but may be a result of hormonal communication between the structures, which would not be present when tissues are separated.

In summary, our study is the first to demonstrate functional endothelial responses in pulmonary vessels in whole lung slices *in situ*. We describe a technique whereby vascular and airway responses can be studied in parallel in a physiologically superior technique. Finally we provide data which suggests that some differences do exist between responses (of airways) in lung slices versus in isolation.

## Competing interests

The author(s) declare that they have no competing interests.

## Authors' contributions

ML completed the experimental work and designed the experimental protocols. JAM conceived the idea of the manuscript and co-designed the experiments and worked closely with ML in the preparation and submission of the manuscript. Other authors contributed equally providing help, guidance and advice.
